# Thermal regimes during overwintering recovery shape microbial network and dissolved organic matter complexity in *Microcystis*-dominated systems

**DOI:** 10.1093/ismejo/wraf227

**Published:** 2025-10-13

**Authors:** Yang Liu, Zongjie Xie, Jia Feng, Shulian Xie, Chao Ma

**Affiliations:** Shanxi Key Laboratory for Research and Development of Regional Plants, School of Life Science, Shanxi University, Taiyuan, Shanxi 030006, China; Shanxi Key Laboratory for Research and Development of Regional Plants, School of Life Science, Shanxi University, Taiyuan, Shanxi 030006, China; Shanxi Key Laboratory for Research and Development of Regional Plants, School of Life Science, Shanxi University, Taiyuan, Shanxi 030006, China; Shanxi Key Laboratory for Research and Development of Regional Plants, School of Life Science, Shanxi University, Taiyuan, Shanxi 030006, China; Institute of Surface-Earth System Science, School of Earth System Science, Tianjin University, Tianjin, Tianjin 300072, China

**Keywords:** overwintering recovery, *microcystis aeruginosa*, microbial assembly, dissolved organic matter, fourier transform ion cyclotron resonance mass spectrometry

## Abstract

The overwintering recovery of *Microcystis aeruginosa* represents a critical but underexplored phase in the seasonal development of cyanobacterial blooms. Although the role of temperature in driving bloom onset is recognized, its effects on microbial assembly and the molecular transformation of dissolved organic matter during reactivation remain insufficiently characterized. In this study, 16S rRNA gene sequencing, excitation-emission matrix fluorescence spectroscopy coupled with parallel factor analysis, Fourier transform ion cyclotron resonance mass spectrometry, and metabolomics were applied to examine how three thermal recovery regimes—constant temperature, gradual warming, and cold-dark preconditioning—shape microbial succession and dissolved organic matter dynamics. Constant temperature accelerated the dispersal limitation of bacterial communities and promoted rapid dissolved organic matter (DOM) turnover, whereas gradual warming and cold-dark preconditioning induced more undominated community structures, and the accumulation of nitrogen- and sulfur-rich DOM compounds. Cold-dark pretreatment notably enhanced the formation of structurally complex, recalcitrant DOM, and delayed microbial reactivation. The network of relationships between microorganisms and dissolved organic matter revealed distinct coupling patterns across treatments, with enhanced microbial processing of aromatic and humic-like molecules occurring under thermal fluctuation or stress. Metabolomic profiling further indicated different physiological adaptation strategies, with stress-linked metabolites enriched under variable-temperature conditions. These findings highlight the mechanistic links between temperature-driven microbial recovery and dissolved organic matter transformation, providing new insights into how winter conditions influence cyanobacterial bloom trajectories in freshwater ecosystems.

## Graphical Abstract

**Graphical Abstract:**
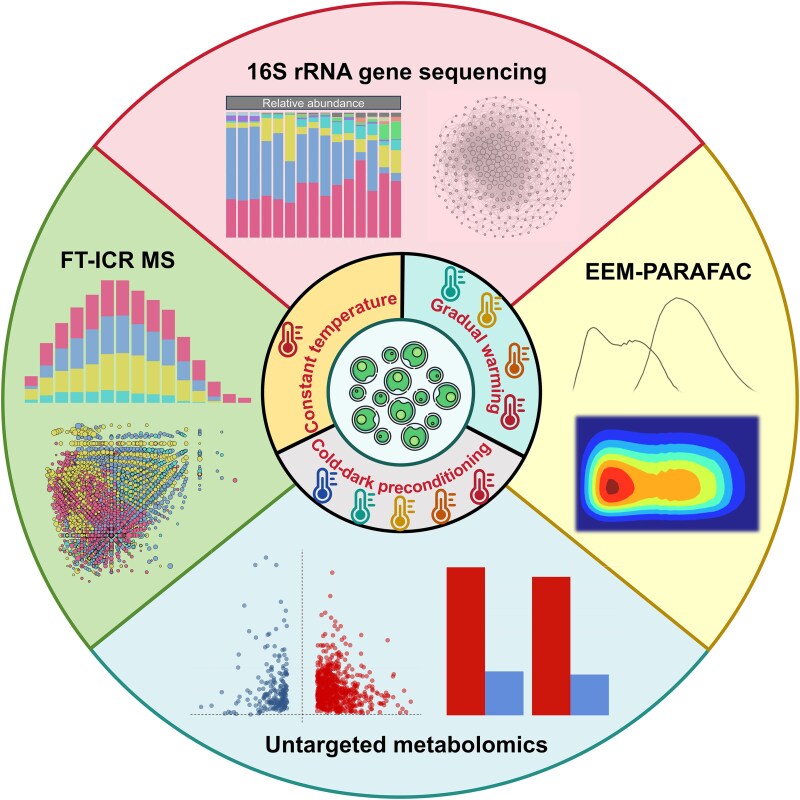

## Introduction

Cyanobacterial harmful algal blooms (CyanoHABs) represent recurrent ecological disturbances in freshwater ecosystems worldwide, with *M. aeruginosa* being one of the predominant bloom-forming species due to its broad tolerance ranges and toxin production capabilities. The seasonal re-emergence of *M. aeruginosa* after overwintering is now recognized as a critical stage in the bloom lifecycle, particularly in temperate lakes and reservoirs [1]. *M. aeruginosa* typically persists in a dormant or low-metabolic state, often within sediment layers, before being reactivated by rising spring temperatures [2, 3]. This metabolic reactivation is highly sensitive to thermal cues, and early onset of spring warming may therefore trigger bloom initiation, increasing the ecological risk of CyanoHAB outbreaks [4].

Dissolved organic matter (DOM) plays a key but often overlooked role in this process. DOM is continuously released by cyanobacteria during growth, senescence, and decay [5]. Algae-derived DOM functions both as a biogeochemical substrate, and as a biological signaling agent, shaping microbial community composition, mediating nutrient cycling, and influencing algal physiology [6–8]. Recent findings indicate that DOM excreted by *M. aeruginosa* can exert autotoxic effects, inhibiting conspecific growth, and thereby establishing a feedback loop in bloom regulation [5]. Through the processing of algal-derived DOM, heterotrophic bacteria transform labile compounds into more recalcitrant forms—recalcitrant dissolved organic matter (RDOM)—that persist in aquatic systems and contribute to long-term carbon storage [9, 10]. Laboratory incubations simulating *Microcystis* bloom degradation have further demonstrated that a fraction of low-molecular-weight DOM resists microbial degradation and accumulates as stable RDOM [11]. Environmental drivers such as light and temperature strongly affect both microbial community structure and DOM composition [12, 13]. Light availability, in particular, modulates bacterial network complexity and metabolic functions during cyanobacterial bloom senescence and DOM turnover. Likewise, temperature fluctuations during overwintering can substantially influence subsequent bloom development by enhancing the regrowth potential of *M. aeruginosa* under elevated spring conditions [4, 14].

Recent studies have increasingly recognized that DOM dynamics and microbial community succession are tightly interconnected through complex ecological networks [12]. However, many investigations of overwintering recovery have examined these processes in isolation, focusing either on microbial assembly mechanisms or on DOM compositional changes, without concurrently evaluating their mechanistic coupling under different postwinter warming scenarios [5, 15, 16]. This gap constrains the ability to clarify how microbial metabolic pathways restructure DOM pools during bloom reactivation. Studies have shown that stable postdormancy conditions tend to promote deterministic selection of microbial taxa [17], whereas environmental variability and legacy effects from prior cold stress enhance stochasticity in assembly processes [18]. Similarly, rapid warming has been shown to accelerate the turnover of labile DOM components [19], while thermal fluctuations or delayed warming can favor the production of nitrogen- and sulfur-enriched RDOM [8, 20].

Despite the recognized importance of these interactions, studies specifically addressing the associations between microbial succession and DOM molecular transformations during the overwintering recovery of *M. aeruginosa* remain scarce. Particularly, the combined effects of temperature regimes—such as constant temperature, gradual warming, and cold-dark preconditioning—on microbial recovery and DOM dynamics have not been systematically assessed. To address this gap, a controlled laboratory experiment was conducted using natural water from the Fen River and pure strains of *M. aeruginosa* PCC 7806. The objectives were to: (i) determine how bacterial community assembly trajectories vary across recovery scenarios; (ii) evaluate DOM compositional dynamics at both optical and molecular levels; and (iii) investigate the coupling between bacterial succession and DOM transformation. This integrative approach provides mechanistic insights into bacteria-DOM interactions during cyanobacterial recovery and the early stages of bloom formation.

## Materials and methods

### Microalgal cultivation, environmental water sampling, and experimental design

The *M. aeruginosa* PCC 7806 used in this study was sourced from the Freshwater Algae Culture Collection at the Institute of Hydrobiology, Chinese Academy of Sciences. Initial cultures were established in 500 ml flasks containing BG11 medium. Cultivation was carried out under controlled conditions: temperature maintained at 25°C, illumination at 100 μmol photons m^−2^.s^−1^ with a 12:12 h light–dark photoperiod, and continuous agitation at 100 rpm using an orbital shaker. A random placement strategy was employed to ensure consistent conditions across all flasks. Cell growth was monitored quantitatively with a hemocytometer and qualitatively using a BX51 light microscope (Olympus, Japan). Fresh river water was collected from the Fen River in Shanxi Province (112.52°E, 37.71°N) in October 2024 at a depth of 0–50 cm and transported to the laboratory within 4 h. Water quality parameters are presented in Table S1. The water was prefiltered through 0.45 μm membranes to remove zooplankton and large particles, while retaining the indigenous microbial community, which was subsequently introduced into the experimental system. The experiment was conducted in a 2 l conical flask containing 1.8 l of mixed culture (*M. aeruginosa* PCC 7806 plus filtered river water). A 12:12 light–dark cycle was maintained at a light intensity of 100 μmol·m^−2^.s^−1^. Exponentially growing *M. aeruginosa* PCC 7806 cultures, with an initial optical density of 0.038–0.041, were allocated into three temperature recovery treatments, each with three biological replicates (*n* = 3). In the constant temperature group (G1), cultures were directly incubated at 25°C for 20 days. In the gradual warming group (G2), cultures were initially incubated at 10°C, and the temperature was increased by 5°C every five days until reaching 25°C over 20 days. In the cold-dark preconditioning group (G3), cultures were maintained in the dark at 4°C for 10 days to simulate winter dormancy, then re-inoculated, and incubated at 10°C, followed by stepwise warming at 5°C increments every five days for 20 days, reaching 25°C. All glass flasks were pretreated at 400°C for 5 h in a muffle furnace to eliminate potential carbon contamination.

### Extraction and quantification of community-derived genomic DNA

Genomic DNA was extracted from community samples using the MagBind Soil DNA Kit (Omega, M5635-02, USA) according to the manufacturer’s instructions. DNA concentration and purity were determined with a Qubit 4.0 fluorometer (Thermo Fisher Scientific, USA) to ensure suitability for downstream applications. Details of PCR amplification, library construction, quantification, and high-throughput sequencing are provided in the Supplementary Information (Text S2).

### Spectroscopic analysis of dissolved organic matter

DOM samples were filtered through 0.45 μm polycarbonate membranes (Millipore) into precombusted amber vials and stored at −20°C in the dark until analysis. Excitation-emission matrix (EEM) spectroscopy was performed using a Hitachi F-7100 fluorescence spectrophotometer (Tokyo, Japan). Calibration was conducted prior to scanning. Instrument parameters were: PMT voltage, 700 V; excitation range, 200–450 nm; emission range, 250–550 nm; scan speed, 12 000 nm/min; and excitation/emission slit widths, 5 nm. The fluorescent properties of DOM were assessed using EEM spectroscopy combined with parallel factor analysis (PARAFAC) modeling. Component identification and validation were carried out using the OpenFluor database, split-half validation, and residual analysis. Spectral indices, including the fluorescence index (FI), biological index (BIX), freshness index (β/α), and humification index (HIX), were calculated following established protocols [21, 22]. Definitions and descriptions of identified fluorescent components are provided in Supplementary Table S2.

### Solid-phase extraction and FT-ICR MS analysis of DOM samples

DOM was extracted by solid-phase extraction (SPE) and subsequently characterized by high-resolution Fourier transform ion cyclotron resonance mass spectrometry (FT-ICR MS). Prior to extraction, all glassware was decontaminated through sequential acid washing (HCl), ultrapure water rinsing, and thermal combustion at 400°C for 5 h to eliminate trace organic residues. SPE-DOM samples (100 ml) were filtered through 0.45 μm polycarbonate membranes (Millipore), collected in acid-cleaned amber glass vials, and stored at −20°C in the dark until analysis. To minimize interference from inorganic salts during ionization, a desalting step was performed following previously described [23].

SPE was conducted using Bond Elut PPL cartridges (200 mg, 3 ml; Agilent Technologies). Cartridges were preconditioned with liquid chromatography-mass spectrometry grade (LC–MS-grade) methanol and acidified ultrapure water (pH = 2) prior to sample loading. Filtrates were passed through the cartridges by gravity using Teflon tubing. After extraction, cartridges were dried in the dark under high-purity nitrogen, and DOM was eluted with LC–MS-grade methanol. Eluates were stored at −20°C until instrumental analysis. Mass spectrometric analysis was performed using a 7.0 T Solarix 2XR FT-ICR MS (Bruker Corp., Billerica, MA, USA) equipped with an electrospray ionization (ESI) source. The polar nature of DOM facilitates ionization under ESI conditions, enabling detection of heteroatom-enriched molecular species in complex matrices. Spectra were acquired across an *m/z* range of 100–900 with a minimum signal-to-noise ratio of six. Mass calibration, formula assignment, and data analysis followed the protocols outlined by previously published method [24], using Compass Data Analysis software (Bruker) alongside an in-house batch-processing tool (mass error tolerance ≤500 ppb). Elemental constraints for molecular formula assignment were defined as C_5–43_, H_3–61_, O_2–18_, N_0–2_, and S_0–1_, consistent with prior studies [25, 26]. High assignment accuracy was maintained with a mass precision better than 0.2 ppm. Further technical specifications and parameter thresholds are provided in Supplementary Table S3.

### Untargeted metabolomics analysis via LC–MS

Untargeted metabolomic profiling of microbial samples was performed by Shanghai Sangon Biotech Co., Ltd. Detailed protocols are provided in Supplementary Text S3.

### Statistical analysis

All statistical analyses were conducted in R (version 4.2.2). Data visualization and multivariate statistical computing were performed using a combination of packages from the tidyverse suite (e.g. “ggplot2”, “dplyr”, and “tidyr”) as well as specialized tools for ecological, compositional, and omics data. Microbial alpha diversity indices were calculated using the “vegan” and “microbiome” R packages. Linear regression models were fitted to examine diversity trajectories over time, with coefficients of determination (*R*^2^) and *P* values obtained using the “lm” function. Bray–Curtis dissimilarity matrices were computed using “vegdist” (from “vegan”), and principal coordinates analysis was conducted to assess beta diversity patterns. To differentiate deterministic from stochastic processes shaping microbial assembly, the beta nearest taxon index (βNTI) was calculated with the “picante” package. Values of |βNTI| > 2 were interpreted as indicative of deterministic processes, while values between −2 and + 2 suggested stochastic assembly mechanisms. Co-occurrence networks of bacterial operational taxonomic units (OTUs) were constructed using Spearman’s correlation matrices, retaining significant associations (|ρ| > 0.6, false discovery rate-adjusted *P* < .01). Network topology parameters were calculated using “igraph” and “psych”, and visualizations were rendered with “ggraph” and “tidygraph”. Mantel tests were performed to assess correlations between dissimilarity metric of microbial community composition, DOM molecular features, and optical indices using the “mantel” function in “vegan”. Correlation network analysis was performed using the OmicStudio tools [27]. For interaction networks (OTU-DOM compounds), edge-weighted significance (*P* < .05) was used to construct visual matrices in Gephi.

## Results

### Bacterial community dynamics, assembly processes, and functional shifts under different thermal regimes

**Figure 1:**
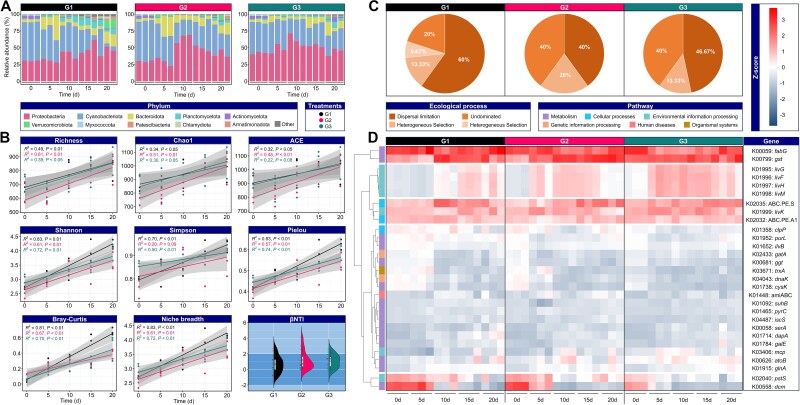
Microbial community succession, diversity trajectories, ecological assembly processes, and functional gene responses during temperature-mediated recovery of *M. aeruginosa*. (A) Stacked bar plots showing temporal shifts in bacterial community composition at the phylum level across three thermal recovery treatments: G1 (constant temperature at 25°C), G2 (gradual warming from 10 to 25°C), and G3 (cold-dark preconditioning followed by progressive warming). Samples were collected at 0, 5, 10, 15, and 20 days, and community composition was determined via 16S rRNA gene sequencing. Only dominant phyla (> 1% relative abundance) are displayed. (B) Changes in alpha diversity metrics over time, including the Richness, Chao1, ACE, Shannon, Simpson, and Pielou indices. Linear regression lines with 95% confidence intervals (shaded areas) are shown for each treatment group. *R^2^* values and statistical significance (*P-*values) are indicated. The Bray–Curtis dissimilarity and niche breadth also increased over time, suggesting community diversification. The violin plots on the bottom right show β-nearest taxon index (βNTI) distributions, which indicate stochastic dominance in community assembly processes (−2 < βNTI < +2). (C) Relative contributions of ecological assembly processes. (D) Heatmap of normalized KEGG orthologs involved in metabolism, transport, and stress responses. All data are based on biological triplicates. For gene abbreviations and detailed annotations, see Supplementary Table S4.

Microbial communities exposed to different thermal regimes exhibited distinct patterns in taxonomic composition, diversity trajectories, ecological assemblies, and functional gene profiles over the 20-day incubation period (Fig. 1). At the phylum level, all groups were consistently dominated by Proteobacteria and Bacteroidota, though relative abundances and temporal dynamics varied markedly among treatments (Fig. 1A). In the constant temperature group (G1), the relative abundance of Proteobacteria increased steadily, reaching ~50% by Day 20, while the abundance of Cyanobacteria decreased. Under gradual warming (G2), Proteobacteria also increased but at a slower rate, with Cyanobacteriota declining after day 10. Cold-dark preconditioning (G3) delayed community shifts, with Proteobacteria maintaining a high relative abundance throughout.

Alpha diversity indices, including Richness, Chao1, ACE, Shannon, Simpson, and Pielou, increased significantly across all treatments (Fig. 1B), reflecting dynamic microbial turnover and diversification. G1 showed the steepest increases in Richness (*R*^2^ = 0.49, *P* < .01) and Shannon index (*R*^2^ = 0.83, *P* < .01), consistent with rapid community development under stable warm conditions. In comparison, G3 exhibited a more gradual increase, likely reflecting delayed reactivation following cold-dark stress. Community dissimilarity, assessed by Bray–Curtis dissimilarity metric, also increased over time, with the fastest divergence observed in G1. Concurrently, niche breadth expanded in all groups, indicating greater ecological and functional heterogeneity. βNTI values across G1, G2, and G3 predominantly fell within −2 < βNTI < +2, indicating that community assembly was largely governed by stochastic processes. Neutral community model results, with progressively improved fit over time, further supported the predominance of random processes (Fig. S2).

The relative contributions of ecological processes to microbial community assembly varied across treatments (Fig. 1C). In G1, succession was predominantly governed by dispersal limitation (60%), followed by heterogeneous selection (13.33%), undominated processes (20%), and a minor contribution from homogeneous selection (6.67%). Under gradual warming (G2), dispersal limitation and undominated processes each accounted for 40%, with heterogeneous selection contributing 20%. In G3, dispersal limitation remained dominant (46.67%), while undominated processes (40%) and heterogeneous selection (13.33%) also played notable roles. |βNTI| > 2 indicates that deterministic processes (e.g. selection) govern assembly, whereas |βNTI| < 2 indicates the dominance of stochastic processes (e.g. dispersal limitation). Across all treatments, the majority of |βNTI| values were < 2 (Fig. 1B), indicating that stochastic processes predominated during bacterial community recovery. Dispersal limitation was the most influential stochastic process in the constant temperature treatment.

Distinct temporal patterns in functional gene abundance were observed across the different treatments (Fig. 1D). In G1, genes associated with amino acid transport and metabolism (e.g. *livG*, *livF*, *livH*, and *livM*), and lipid metabolism (*fabG*) showed consistently stable and high expression levels throughout the incubation period relative to other genes in the same treatment, indicating active microbial metabolic processes under stable warm conditions. In contrast, G2 exhibited more variable gene abundances, with transient increases in amino acid metabolism and stress-related genes (*clpP* and *trxA*), suggesting a gradual metabolic activation during progressive warming. In G3, many functional genes displayed a delayed but pronounced enrichment over time, particularly in genes involved in nitrogen metabolism (*glnA*, *cysK*, and *gatA*) and redox processes (*ggt*, *iscS*, and *trxA*), reflecting metabolic adjustments associated with reactivation after cold-dark preconditioning. Overall, the functional gene profiles indicated that temperature strongly influenced microbial metabolic activity and pathway regulation during cyanobacterial recovery.

### Co-occurrence network structure and topological properties

**Figure 2:**
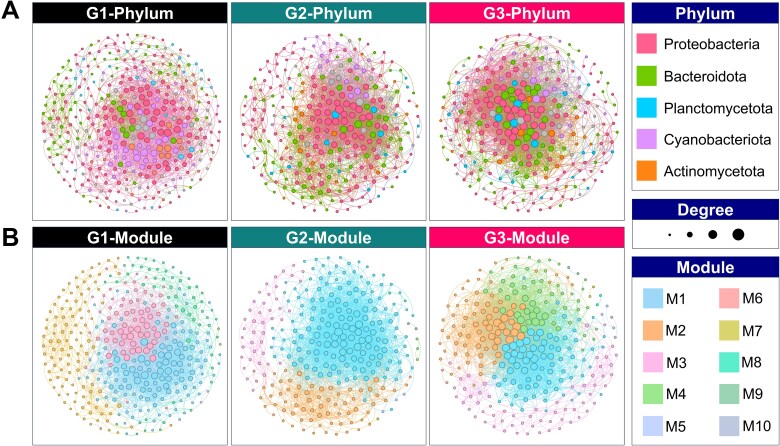
Co-occurrence network structures of bacterial communities under different treatments of *M. aeruginosa*. (A) Taxonomic affiliations of network nodes at the phylum level. Node colors represent major phyla, and node size corresponds to degree. (B) Modular organization of each network is based on community detection algorithms. Modules (M1–M10) are color-coded and indicate subnetwork clusters with potential ecological coherence.

To investigate microbial interactions under varying thermal regimes, co-occurrence networks were constructed for each treatment group. The networks differed in topological complexity, modular structure, and interaction patterns (Fig. 2, Tables S5 and S6). The network of G1, representing the constant temperature condition, comprised the highest number of nodes (368) but the fewest edges (2197), resulting in the lowest network density (0.033). It exhibited moderate modularity (0.42), with three dominant modules (M1: 36.41%, M2: 28.26%, and M3: 18.21%) accounting for over 82% of the network composition. The average clustering coefficient was 0.43, and the average degree was 11.94, indicating moderate local connectivity. The proportion of positive correlations (87.80%) was highest in G1, suggesting that microbial interactions were predominantly cooperative under stable environmental conditions. In contrast, the G2 network, which underwent gradual warming, showed higher connectivity (average degree = 17.52; 2462 edges), a shorter network diameter (10), and a denser structure (density = 0.06). The network was heavily dominated by a single module (M1: 56.94%), suggesting that the progressive temperature increase had a strong homogenizing effect. The average clustering coefficient (0.50) was higher in G2 than in G1, while the proportion of negative correlations was also higher (25.95%), implying that competitive or antagonistic interactions exerted a greater influence. The G3 network, which experienced a cold-dark pretreatment followed by rewarming, was the most complex in terms of connectivity, with the highest average degree (23.74) and number of edges (3300), along with the highest density (0.09) and clustering coefficient (0.55). Despite having a similar network diameter to G2 (10), the G3 network exhibited greater modularity (0.28), with four primary modules (M1–M4) contributing relatively evenly (28.06%–21.94%). This indicated greater niche partitioning and ecological differentiation in G3. The proportion of negative correlations was highest in G3 (31.36%), suggesting that competitive dynamics and selective pressures were intensified during recovery from environmental stress.

### Dynamics of DOC concentrations and DOM fluorescence characteristics

**Figure 3:**
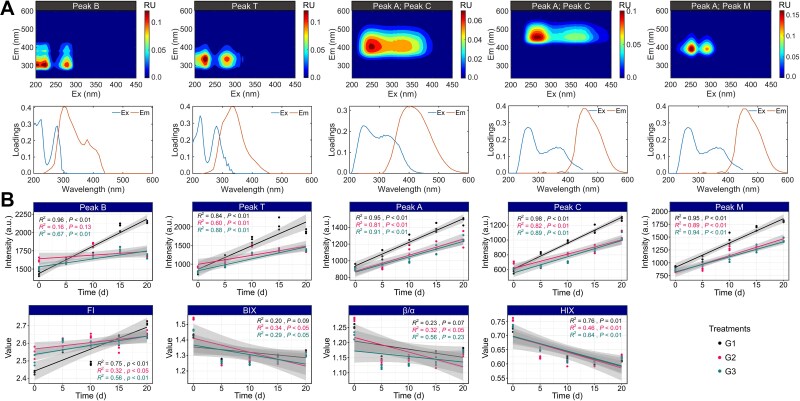
Dynamics of fluorescent DOM components and spectral indices under different treatments of *M. aeruginosa*. (A) EEM-PARAFAC analysis identified five fluorescent DOM components, including peaks B, T, A, C, and M. The upper panels show fluorescence contour plots; the lower panels show excitation and emission loadings for each component. (B) Temporal trends of peak intensities and spectral indices (FI, BIX, β/α, and HIX) under three treatments: *R*^2^ values indicate the strengths of temporal trends.

Dissolved organic carbon (DOC) concentrations exhibited rapid declines during the initial 10 days of incubation in all treatments (Fig. S3), decreasing from ~4.0 mg/l to below 1.6 mg/l. While the general decreasing trend was consistent across treatments, G3 exhibited slightly lower DOC concentrations at later stages compared to G1 and G2. The fluorescence characteristics of DOM were analyzed using EEM-PARAFAC. Five distinct fluorescent components were identified, corresponding to protein-like peaks (B and T), humic-like peaks (A and C), and a microbial humic-like peak (M) (Fig. 3). Their spectral profiles and corresponding loadings were consistent with previously characterized DOM components in aquatic systems (Table S2).

All five fluorescent peaks exhibited increases in fluorescence intensity over time, with clear differences in rate and magnitude among treatments. Fluorescence intensities increased most rapidly under constant temperature conditions (G1), particularly for protein-like components. Peak B (tyrosine-like) showed a strong linear increase (*R*^2^ = 0.96, *P* < .01), suggesting elevated microbial activity and fresh DOM release. Similarly, peak T (tryptophan-like) and humic-like (peaks A and C) displayed substantial temporal increases (*R*^2^ = 0.84–0.98, *P* < .01), indicating active transformation and accumulation of humified material. In contrast, G3 showed lower initial intensities and more gradual increases across all components, particularly peaks B and T (*R*^2^ = 0.67 and 0.88, respectively), reflecting delayed DOM production likely due to reactivation stress following cold-dark incubation. G2 presented intermediate patterns, aligning with its transitional thermal regime. Microbial humic-like peak M increased steadily in all treatments, with G1 again exhibiting the most pronounced trend (*R*^2^ = 0.95, *P* < .01), followed by G2 and G3 (*R*^2^ = 0.89 and 0.94, *P* < .01, respectively), indicating progressively intensifying microbial processing of DOM across treatments.

The FI increased significantly over time in all groups (*R*^2^ = 0.75 in G1, 0.32 in G2, and 0.56 in G3). The highest value in G1 suggested that the microbial contribution to DOM pools was enhanced under stable warm conditions. The BIX was also notably higher in G1 and G3 (*R*^2^ = 0.20–0.34) than in G2. The β/α ratio showed a slight decreasing trend in G1 and G2 (*R*^2^ = 0.23–0.32) but remained relatively stable in G3 (*R*^2^ = 0.56, *P* < .05). HIX, indicative of DOM recalcitrance, showed a consistent decreasing trend across all groups. Bray–Curtis dissimilarity analysis revealed that DOM composition diverged fastest in G1, moderately fast in G3, and slowest in G2 (Fig. S4). Increases in dissimilarity were observed for all fluorescent peaks and optical indices in G1 and G3, whereas G2 showed weaker changes, particularly for peak B (r = 0.15, *P* = .079). These findings indicated that *Microcystis* was affected by differences in temperature regime, which significantly influenced the conversion rate of DOM molecules.

### Molecular-level transformations of DOM characterized by FT-ICR MS

**Figure 4:**
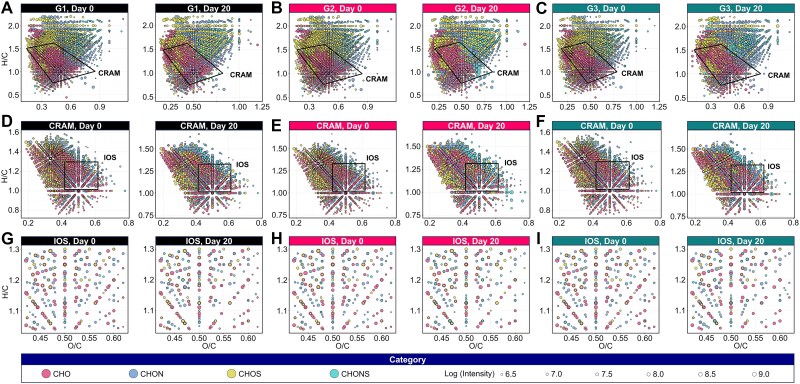
Molecular composition and transformation of DOM under different treatments as revealed by FT-ICR MS. Van Krevelen diagrams (H/C vs. O/C) for DOM molecules in treatments G1, G2, and G3 at Day 0 and Day 20. Each point represents a molecular compound classified by heteroatom class (CHO, CHON, CHOS, and CHONS); point size corresponds to log-transformed relative intensity. (A–C) Broad-scale molecular distributions highlighting the carboxyl-rich alicyclic molecules (CRAM) region (outlined by black polygons). (D–F) Expanded regions highlighting island of stability (IOS) molecules, marked by black squares. (G–I) High-resolution views of IOS zones for detailed comparisons of composition shifts.

The molecular composition and chemical characteristics of DOM showed distinct time- and treatment-specific changes, as revealed by FT-ICR MS analysis (Fig. 4, Table 1). Van Krevelen plots indicated the consistent presence of carboxyl-rich alicyclic molecules (CRAMs) and isooctyl-substituted (IOS)-type compounds across all samples, with varying densities and elemental compositions (Fig. 4). From Day 0 to Day 20, all treatments exhibited expansions of CRAM compounds, especially G1 and G3, suggesting progressive accumulation of carboxyl-rich, recalcitrant molecules. IOS components were also enriched, particularly in G2 and G3, reflecting microbial contributions to stable DOM fractions. CHO compounds remained the dominant class throughout the study (~32%–34%), while CHON increased slightly in G1 and G2 but decreased in G3 by day 20. Carbon-hydrogen-oxygen-sulfur (CHOS) and carbon-hydrogen-oxygen-nitrogen–sulfur (CHONS) fractions increased in G2 and G3, especially CHONS in G2 (from 8.95% to 13.14%) and in G3 (from 9.89% to 10.92%), indicating that sulfur- and nitrogen-rich compounds accumulated under variable or stressful conditions. The average nominal oxidation state of carbon (NOSC) remained stable (−0.38 to −0.44), while H/C and O/C ratios showed slight increases, indicating mild oxidative transformation. Aromaticity index (AI_mod_) and double bond equivalents (DBEs)-related parameters remained largely unchanged, though G2 showed a minor increase in DBE/C and G3 displayed relatively high DBE/H values, suggesting a shift toward slightly more unsaturated structures. Aromatic compound percentages (AI_mod_ > 0.5) remained low across all treatments (<7%), while highly unsaturated phenolic-like molecules dominated (~53%–57%), indicating a prevalence of aquatic humic substances. Aliphatic compounds (AI_mod_ ≤ 0.5, H/C ≥ 1.5) decreased in G3 (from 34.38% to 18.28%), suggesting transformation toward more aromatic or unsaturated compounds during recovery. All groups showed reductions in average molecular weight after 20 days. Mass ranges indicated a general redistribution from high (> 450 *m/z*) to medium (300–450 *m/z*) fractions, particularly in G1 and G2.

**Table 1:** Molecular composition and structural characteristics of DOM under different treatments.

		CHO (%)	CHON (%)	CHOS (%)	CHONS (%)	NOSC	H/C	O/C	AI_mod_	DBE/C	DBE/O	DBE/H	DBE	△*G*^o^_Cox_	MW	Polycyclic aromatic compounds (AI_mod_ > 0.67) (%)	Aromatic compounds (0.5 < AI_mod_ ≤ 0.67) (%)	Highly unsaturated and phenolic compounds (AI_mod_ ≤ 0.5, H/C < 1.5) (%)	Aliphatic compounds (AI_mod_ ≤ 0.5, H/C ≥ 1.5) (%)	150 < *m/z* ≤ 300 (%)	300 < *m/z* ≤ 450 (%)	*m/z* > 450 (%)
G1	Day 0	33.99	28.11	28.97	8.93	−0.44	1.39	0.44	0.14	0.37	0.92	0.31	7.59	72.88	440.51	1.26	5.22	56.15	37.37	16.27	39.37	44.36
	Day 20	32.11	29.41	27.02	11.46	−0.44	1.40	0.45	0.13	0.37	0.91	0.30	7.34	72.76	436.15	1.59	4.94	53.60	39.87	17.23	37.48	45.28
G2	Day 0	32.69	28.21	30.15	8.95	−0.42	1.39	0.45	0.15	0.38	0.91	0.32	7.52	72.27	434.44	1.55	5.54	56.24	36.67	16.87	39.64	43.49
	Day 20	30.69	30.75	25.42	13.14	−0.43	1.40	0.45	0.14	0.37	0.92	0.31	7.14	72.48	420.77	1.96	5.38	52.54	40.11	18.28	41.46	40.26
G3	Day 0	33.16	29.18	27.77	9.89	−0.38	1.36	0.44	0.16	0.39	0.93	0.33	7.60	71.00	426.65	1.68	6.35	57.59	34.38	17.66	41.19	41.15
	Day 20	31.98	24.85	30.57	12.60	−0.44	1.40	0.44	0.15	0.37	0.94	0.31	7.29	72.96	425.56	1.70	5.23	54.09	38.98	18.28	39.17	42.54

Parameters include elemental group proportions (CHO, CHON, CHOS, and CHONS), molecular ratios (H/C, O/C, DBE/C, DBE/O, and DBE/H), nominal oxidation state of carbon (NOSC), double bond equivalents (DBE), modified aromaticity index (AI_mod_), Gibbs free energy of carbon oxidation (△*G*^o^_Cox_), and molecular weight (MW).

**Figure 5:**
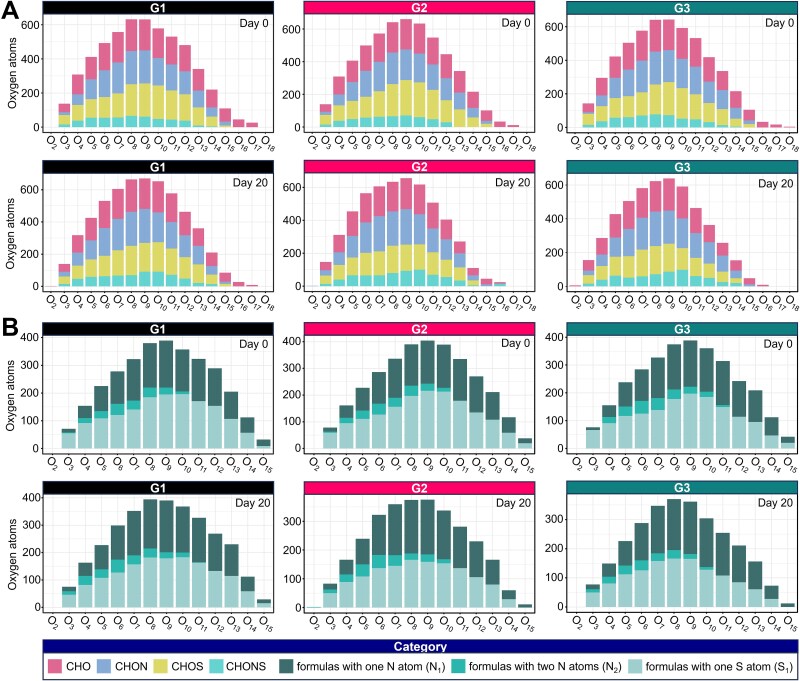
Oxygen atomic distribution patterns of DOM molecular formulas under different treatments. (A) Elemental distributions of oxygen atoms (O_x_) in DOM compounds at Day 0 and Day 20 across three treatments. Bars are stacked by elemental class: CHO, CHON, CHOS, and CHONS. (B) Distribution of nitrogen and sulfur atoms in DOM formulas containing heteroatoms. Bars indicate the number of formulas with one nitrogen atom (N_1_), two nitrogen atoms (N_2_), and one sulfur atom (S_1_).

The molecular composition of DOM, categorized by heteroatom class (Fig. 5). In all three treatments, molecular formulas were predominantly distributed from O_8_ to O_12_, with normal distributions observed on both day 0 and day 20. CHO and CHON compounds were most abundant, followed by CHOS and CHONS, with minimal variation within each group. However, G3 exhibited a slightly higher abundance of CHONS-containing formulas on day 20, suggesting that sulfur- and nitrogen-bearing DOM was enriched during recovery from cold-dark conditions. With respect to nitrogen- and sulfur-containing formulas, the majority of DOM molecules contained one nitrogen atom (N_1_) and one sulfur atom (S_1_), while compounds with two nitrogen atoms (N_2_) were less prevalent.

**Figure 6:**
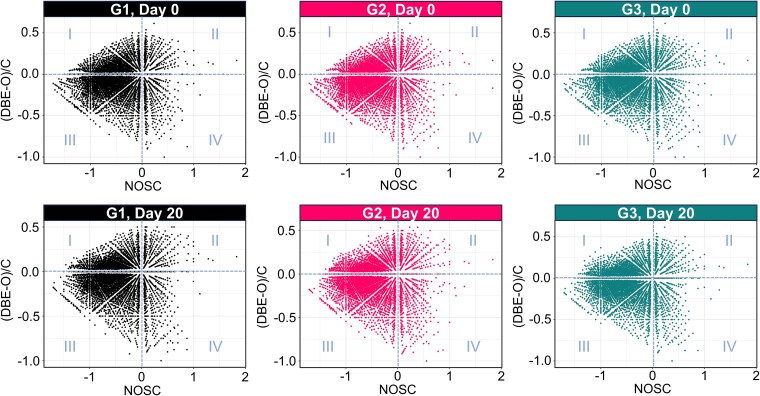
Molecular transformation of DOM under different treatments. The space is divided into four regions: (I) unsaturated and reduced compounds; (II) unsaturated and oxidized compounds; (III) saturated and reduced compounds; and (IV) saturated and oxidized compounds. The (DBE-O)/C ratio served as an indicator of molecular saturation state, where elevated positive values ((DBE-O)/C > 0) reflected increased unsaturation, while negative values ((DBE-O)/C < 0) corresponded to higher saturation levels. Molecular formulas exhibiting positive NOSC values (NOSC > 0) were categorized as oxidized species, whereas negative values (NOSC < 0) identified reduced compounds. Neutral redox states were specifically associated with molecular formulas demonstrating NOSC values (NOSC = 0).

The NOSC-(DBE-O)/C framework revealed that DOM was dominated by reduced and saturated molecules (region III, accounting for ~47%–51%) (Fig. 6, Table S7). In G1 under constant thermal conditions, the molecular distribution remained stable over time, reflecting limited redox restructuring. Gradual warming in G2 induced moderate oxidation and unsaturation, as evidenced by decreases in region III and increases in regions II and IV. In contrast, G3 exhibited a rise in the reduced-unsaturated (region I) and reduced-saturated (region III) fractions, suggesting thermodynamically stable compounds were being reassembled during recovery from cold-dark stress.

### Correlations between microbial taxa and DOM molecular characteristics

**Figure 7:**
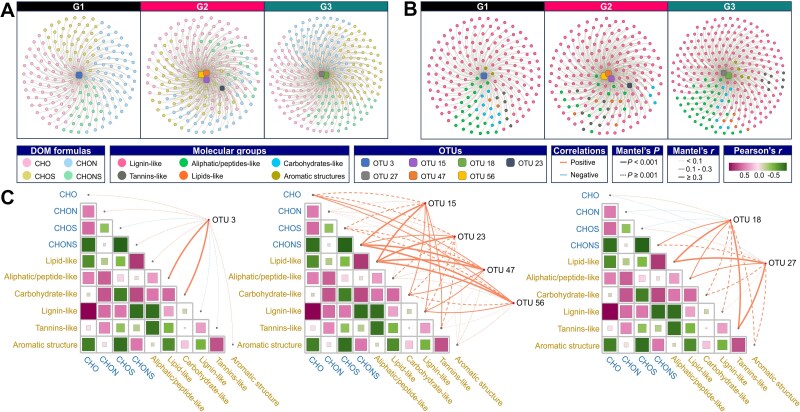
Correlations between bacterial OTUs and DOM molecular features under different treatments. (A) Interaction networks linking OTUs (squares) with DOM molecular features (circles) in each treatment. Molecular features are color-coded by elemental composition (CHO, CHON, CHOS, and CHONS). (B) Network plots showing associations between specific OTUs and DOM molecular groups. (C) Correlation matrices and Mantel test results between DOM groups and OTUs. Node–edge overlays highlight representative OTUs with strong positive linkages (i.e. OTU 3 in G1, OTUs 15/23/47/56 in G2, OTUs 18/27 in G3), suggesting enhanced coupling between microbial taxa and specific DOM classes under different thermal regimes. Solid edges indicate significant Mantel test results (*P* < .001); dashed edges indicate nonsignificance.

To elucidate the relationships between microbial taxa and DOM compositions, two complementary analytical approaches were employed (Fig. 7). Network analysis (Fig. 7A and B) was used to visualize specific co-occurrence patterns, and direct correlations between individual OTUs and particular DOM molecules. The results revealed markedly different association patterns across thermal regimes: only a single OTU showed significant correlations in G1, whereas G2 and G3 exhibited denser and more complex networks, with multiple OTUs (e.g. OTUs 15, 23, 47, and 56 in G2; OTUs 18 and 27 in G3) showing strong positive associations with various DOM molecules, particularly nitrogen- and sulfur-containing compounds. This analysis revealed both the structure and the sign (positive/negative) of fine-scale associations. In contrast, a Mantel test was applied to assess the overall correlation between the complete microbial community composition and the broader DOM molecular profiles (Fig. 7C). This test provided a statistical measure of global, community-level coupling between the entire microbial assemblage and the total DOM pool, rather than individual pairwise relationships. While the network analysis highlighted specific OTU-DOM links that varied in complexity across treatments, the Mantel test confirmed that these relationships collectively resulted in a significant overall association between community structure and DOM composition in each treatment, with varying strength and specificity.

### Differential metabolite profiles across treatments

**Figure 8:**
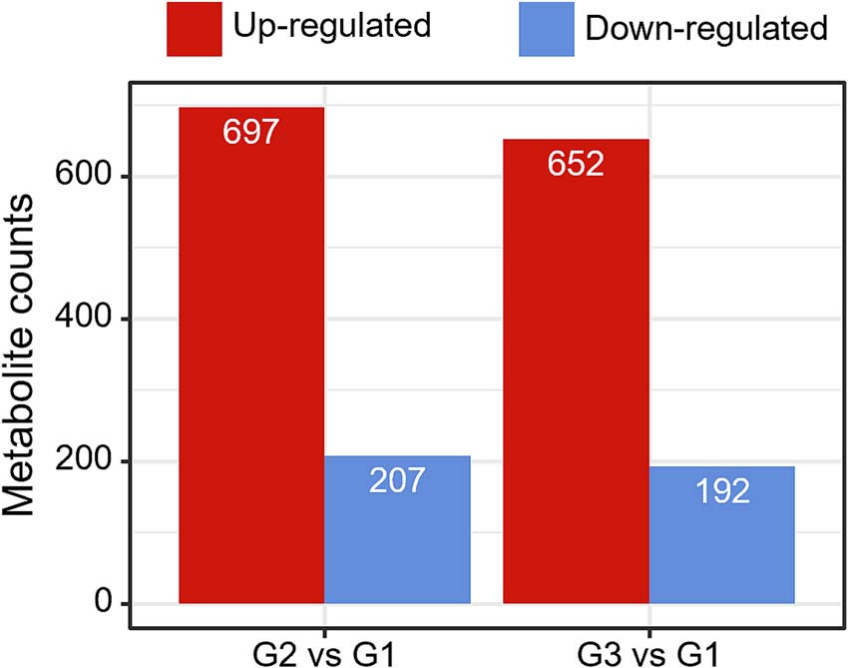
Metabolic counts between different treatment groups. Bar plot showing the number of significantly upregulated (red) and downregulated (blue) metabolites in pairwise comparisons of G2 vs. G1 and G3 vs. G1 based on untargeted metabolomics.

Untargeted metabolomics analysis revealed substantial shifts in intracellular metabolic compositions across treatments (Fig. 8, Table S8 and S9). Compared to G1, both G2 and G3 exhibited metabolic reprogramming, as indicated by the number of differentially regulated metabolites (Fig. 8). A total of 904 differential metabolites were identified in G2 vs G1, including 697 upregulated and 207 downregulated compounds. Similarly, G3 vs G1 yielded 652 upregulated and 192 downregulated features, indicating extensive metabolic activation across both variable-temperature treatments. Multiple upregulated compounds in both G2 and G3 included dipeptides and oligopeptides (e.g. Lys-Ile-Arg-Asp and Thr-Leu-Lys-Lys), phospholipids (e.g. 1,2-Docosahexanoyl-sn-glycero-3-phosphocholine), carotenoids (e.g. Nostoxanthin), and gamma-glutamyl derivatives (e.g. γ-Glutamylglutamate). These metabolites are associated with membrane remodeling, osmotic stress adaptation, photosynthetic pigment biosynthesis, and antioxidant responses, suggesting temperature-induced regulation of both structural and functional metabolic pathways. In addition, several overlapping metabolites—such as γ-Glutamylglutamate, Nostoxanthin, Convolamine, and Ser-Asn-Ser-were consistently upregulated in both G2 and G3, implying shared metabolic signatures in cyanobacterial communities under warming and reactivation conditions. Further, differences in the abundances, and types of upregulated oligopeptides and lipid intermediates suggested that strategies of thermal adaptation and reprogramming differed between treatments.

## Discussion

### Temperature-driven divergence in microbial community assembly and interactions

The thermal regime following overwintering dormancy played a decisive role in shaping bacterial community trajectories during *M. aeruginosa* reactivation. The findings reveal that stochastic processes, rather than deterministic selection, dominated bacterial community assembly across all thermal regimes, as evidenced by |βNTI| values predominantly <2 (Fig. 1B). However, the form of stochasticity varied with thermal history. The constant warming regime (G1) strongly favored dispersal limitation (Fig. 1C), likely because optimal and stable conditions allowed niche-based interactions and population growth to be constrained primarily by physical immigration history. In contrast, the variable thermal regimes (G2 and G3) increased the influence of undominated processes, which may include unpredictable drift or weak dispersal. This shift suggests that environmental fluctuations compounded ecological randomness, making historical contingency and chance events more influential than in the stable environment. Previous studies also observed increased stochasticity in microbial assembly under fluctuating hydrological conditions [28]. The G3 group was influenced more by dispersal limitation compared to G2, suggesting that prior exposure to cold-dark conditions imposed a “legacy effect” on microbial reassembly. Such legacy effects, where previous environmental exposure shapes community plasticity and response capacity, have been increasingly recognized in microbial ecology. For instance, previous research demonstrated that disturbance history influences microbial resilience and community reassembly, particularly after stress events, consistent with our observations [18].

Co-occurrence network analysis further revealed how microbial interactions were restructured under different thermal regimes. Under constant temperature (G1), networks showed moderate modularity and high proportions of positive interactions, implying that cooperative relationships were favored in a stable environment—possibly due to syntrophic exchanges of DOM degradation byproducts. A previous study similarly found that microbial co-occurrence networks under stable conditions tended to display more positive correlations, indicative of metabolic complementarity [29]. In contrast, G3 networks were not only denser and more complex but also showed higher proportions of negative correlations, suggesting intensified competition and niche differentiation. Previous studies reported reduced network complexity under stress [30]. The difference may stem from the timing of network formation.

### Dissolved organic matter transformation pathways and microbial coupling under different temperature regimes

This study provides further evidence that microbial-DOM interactions are tightly coupled with recovery trajectories after overwintering, a link not explicitly addressed in previous studies. Our findings demonstrate that thermal recovery regimes modulate not only microbial community structure but also the capacity of these communities to process and transform DOM. EEM-PARAFAC analyses revealed a clear increase in DOM fluorescence intensity over time, particularly for protein-like (peaks B and T) and humic-like (peaks A and C) components. These trends were most pronounced under constant temperature conditions (G1). In addition, the significant rise in microbial-derived DOM in G1 indicated active microbial reworking of organic substrates. This is consistent with the results of previous research findings, which found that bacterial activity drove rapid shifts in DOM fluorescence and composition, particularly under conditions that favor high microbial turnover [19]. Conversely, the G2 and G3 treatments exhibited delayed increases in both fluorescence intensity and microbial indices, highlighting the physiological costs of reactivation from dormancy. This lag was particularly evident in the tyrosine- and tryptophan-like DOM components, which are closely associated with microbial biomass and turnover.

At the molecular level, FT-ICR MS results revealed that DOM composition shifted toward more recalcitrant forms (e.g. CRAM and IOS molecules) over time. CRAM and IOS were positively associated with HIX and BIX, especially in G3 (Fig. S5). This association implied that humification and transformation processes were enhanced during recovery from cold-dark stress. These results echoed those of previously described [31], which observed that microbial transformation pathways producing chemically stable compounds contributed to long-term carbon sequestration. The accumulation of CHONS and CHOS compounds in G2 and G3 indicated increased microbial production of nitrogen- and sulfur-rich molecules, commonly linked to microbial stress responses or protein degradation. This pattern contrasted with the findings of previously described [32], which emphasized abiotic factors influencing CHONS accumulation; our results underscored the microbial contribution under postdormancy stress conditions. In addition, a previous research noted that stress-induced microbial pathways often favor the production of aromatic compounds that resist microbial degradation, thereby contributing to carbon persistence in aquatic ecosystems [20]. DOM-microbe coupling was strongest in G2 and G3, as revealed by correlation networks and Mantel tests linking specific OTUs with DOM molecular characteristics. For example, CHONS-rich molecules were significantly associated with bacterial taxa involved in complex carbon degradation pathways. This finding supports the previously proposed “microbial carbon pump” framework [8, 9], which posits that microbial metabolic processes convert labile organic matter into stable carbon compounds.

Temperature not only governs cyanobacterial growth rates and bloom dynamics but also modulates exudation, cell lysis, and metabolic byproducts that represent major sources of labile DOM [33, 34]. At elevated and stable temperatures, *Microcystis* cells typically exhibit higher photosynthetic activity, and enhanced exudation of low-molecular-weight carbohydrates and amino acids, thereby fueling rapid microbial utilization and nutrient recycling [35]. Recent research indicates that warming (+4°C) stimulates algal growth and DOC release, yet also enhances bacterial consumption, leading to a net reduction in recalcitrant DOC [36]. By contrast, under variable or suboptimal thermal regimes, cellular stress may increase the release of reactive oxygen species and trigger partial cell lysis, producing more complex RDOM fractions, including humic-like substances [37, 38]. These stress-induced exudates can alter the stoichiometry of DOM available to bacteria and may also include secondary metabolites with allelopathic or autotoxic effects, further constraining bloom development [5, 38]. Thus, the interplay between temperature-driven algal physiology and microbial processing provides a coupled feedback system in which *Microcystis* responses to warming or fluctuating thermal regimes actively shape DOM quantity and quality, and consequently the trajectory of microbial-DOM interactions observed in this study.

### Metabolomic signatures reveal distinct recovery strategies in *M. aeruginosa* PCC 7806

The integration of metabolomic and microbial data provided a holistic view of the physiological adjustments underlying *M. aeruginosa* recovery. Untargeted metabolomics revealed substantial reprogramming in the G2 and G3 treatments, with upregulation of peptides (e.g. Lys-Ile-Arg-Asp), carotenoids (e.g. nostoxanthin), and phospholipids—metabolites known for their roles in stress adaptation, antioxidant defense, and membrane stabilization. These findings are consistent with previously published research [39], demonstrating that environmental stress leads to the accumulation of antioxidant-related metabolites in cyanobacteria as part of a rapid response strategy. Shared metabolites such as γ-glutamylglutamate across G2 and G3 indicated core biochemical strategies employed during reactivation. This metabolite is a precursor in glutathione metabolism and is known to alleviate oxidative stress. The distinct metabolic profiles of G2 and G3 further implied that *M. aeruginosa* tailors its stress responses based on the rate and nature of environmental change. G3 cultures showed signatures enriched in nitrogen-containing osmolytes and aromatic amino acid derivatives, suggesting a metabolic shift toward nitrogen storage and structural stabilization. Recent studies have shown that cyanobacteria accumulate specialized metabolites during prolonged dormancy to support slow but resilient reactivation [40, 41]. In contrast, the G1 group under constant temperature conditions exhibited fewer stress-related metabolites but elevated energy-yielding intermediates (e.g. citric acid cycle components), indicative of rapid growth in the initial stage of an algal bloom. In addition, fast microbial reactivation leads to efficient DOM turnover and early bloom onset, creating a positive feedback loop between microbial metabolism and cyanobacterial proliferation. Previously published research has documented similar dynamics, describing how warming trends promoted earlier and more intense cyanobacterial blooms through enhanced metabolic readiness [42]. Conversely, cold-dark preconditioning in G3 delayed this feedback loop but enhanced metabolic diversity and DOM recalcitrance, potentially stabilizing the microbial loop and altering bloom trajectories. Moreover, the release of complex secondary metabolites—many of which are associated with stress responses or allelopathic activity—may further reshape microbial interactions and, in turn, influence the competitive balance of *Microcystis* within the phytoplankton assemblage [43]. In this way, the imprint of the thermal regime extends beyond growth kinetics to the biochemical framework in which blooms unfold, establishing a feedback that can either promote a rapid, high-intensity bloom under favorable conditions or delay bloom development under a qualitatively different DOM and microbial context, with direct consequences for bloom magnitude and persistence.

### Study limitations

Although this study offers a comprehensive multi-omics perspective on microbial and DOM responses to thermal regimes, several limitations remain. First, laboratory incubations simplify natural conditions by excluding dynamic factors such as light fluctuations, nutrient gradients, hydrodynamics, and biotic interactions. Field-based validations and long-term *in situ* observations are needed to verify these findings under real-world complexity. Second, the study primarily focused on bacterial and cyanobacterial communities, while overlooking eukaryotic microbes, viruses, and higher trophic levels that may influence microbial assembly and DOM processing. Incorporating these components would yield a more complete understanding of microbial food web responses to thermal fluctuations. Third, the short-term scope of this study limits insight into long-term resilience, legacy effects, and the potential for evolutionary adaptation. Long-term studies incorporating repeated thermal disturbances and multigenerational analyses will be essential for assessing cyanobacterial responses to climate-driven thermal variability. Collectively, these limitations highlight the need for experiments with broader temporal, ecological, and molecular frameworks to fully resolve the mechanisms governing microbial resilience and DOM cycling in freshwater systems under global warming.

## Conclusions

This study demonstrates how temperature governs microbial community assembly, functional gene expression, DOM transformation, and metabolic reprogramming during *M. aeruginosa* recovery. Constant temperature conditions promoted rapid microbial succession, metabolic activation, and DOM turnover, facilitating early bloom development. In contrast, gradual warming and cold-dark preconditioning delayed microbial reactivation but enhanced metabolic diversity, reflected in nitrogen- and sulfur-enriched DOM and distinct intracellular metabolite profiles. These findings highlight the sensitivity of microbial-DOM linkages to thermal variability and underscore the importance of considering both community composition and metabolite-level processes when evaluating organic matter transformations. Overall, this work provides a mechanistic basis for understanding bloom dynamics in shallow lacustrine systems.

## Supplementary Material

Supplementary_Information-R4_wraf227

## Data Availability

Sequencing data generated in this study have been deposited in the NCBI database under BioProject accession number PRJNA1280777 and are publicly available. R scripts for statistical analyses are available on GitHub at https://github.com/2766311507-dotcom/R. Metabolomics data reported in this paper have been deposited in the OMIX, China National Center for Bioinformation / Beijing Institute of Genomics, Chinese Academy of Sciences (https://ngdc.cncb.ac.cn/omix: accession no.OMIX012133).
