# Supplementary material for: Thermal regimes during overwintering recovery shape microbial network and dissolved organic matter complexity in Microcystis-dominated systems
Source: ISME J. 2025 Oct 13;19(1):wraf227. doi: 10.1093/ismejo/wraf227 (PMC12596278; doi:10.1093/ismejo/wraf227)
Supplement: Supplementary_Information-R4_wraf227 [file supplementary_information-r4_wraf227.docx]

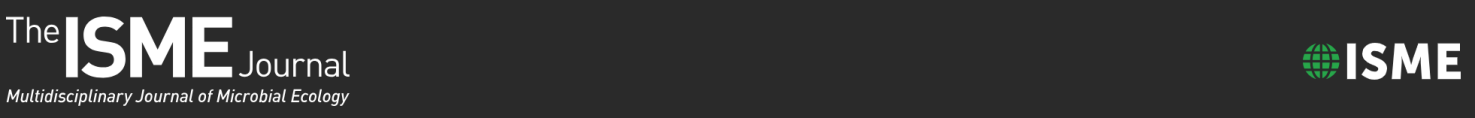


Supplementary material for

**Thermal regimes during overwintering recovery shape microbial network and dissolved organic matter complexity in *Microcystis*-dominated systems**

Yang Liu ^1^, Zongjie Xie ^1^, Jia Feng ^1^, Shulian Xie ^1, *^, Chao Ma ^2, *^

^1^ Shanxi Key Laboratory for Research and Development of Regional Plants, School of Life Science, Shanxi University, Taiyuan 030006, China.

^2^ Institute of Surface-Earth System Science, School of Earth System Science, Tianjin University, Tianjin 300072, China.

**Running title:** Microbial-DOM network of *Microcystis*

^*^ **Corresponding author:**

Shulian Xie: [xiesl@sxu.edu.cn](mailto:xiesl@sxu.edu.cn)

Shanxi Key Laboratory for Research and Development of Regional Plants, School of Life Science, Shanxi University, No. 63, Nanzhonghuan East Street, Taiyuan 030006, China.

Chao Ma: [machao2019@tju.edu.cn](mailto:weiyuqiu@163.com)

Institute of Surface-Earth System Science, School of Earth System Science, Tianjin University, No. 92 Weijin Road, Tianjin 300072, China.

**Contents of this file:**

- **Texts: 4**
- **Figures: 5**
- **Tables: 9**
- **References: 11**

1. **Supplementary Texts**

**Text S1. Determination of water quality parameters**

The concentrations of chemical oxygen demand (COD), total phosphorus (TP), ammonium nitrogen (NH₄-N), and nitrate nitrogen (NO₃-N) were determined by UV-visible spectrophotometry (TU-1810, Persee, Beijing, China). COD was analyzed via the alkaline potassium permanganate method. TP was measured using the phosphomolybdenum blue method according to a previously published method [1]. NH₄-N was quantified with the sodium salicylate method (LOD: 0.03 μmol/L), while NO₃-N was assessed using the cadmium reduction column method (LOD: 0.01 μmol/L) [2]. Dissolved organic carbon (DOC) concentrations were determined using a TOC-L analyzer (Shimadzu, Japan).

**Text S2. PCR amplification and sequencing of bacterial 16S rRNA gene**

Genomic DNA was first quantified using a Qubit 4.0 fluorometer (Thermo Fisher Scientific) and its integrity assessed with an Agilent 2100 Bioanalyzer (Agilent Technologies, USA) to ensure suitability for high-throughput sequencing. Amplification of the bacterial 16S rRNA gene targeted the hypervariable V3–V4 region using PAGE-purified universal primers: forward 341F (CCTACGGGNGGCWGCAG) and reverse 806R (GACTACHVGGGTATCTAATCC). PCR reactions were prepared in a 30 μL final volume containing 2 μL of template DNA (10 ng/μL), 1 μL of each primer (10 μM), and 2× Hieff Robust PCR Master Mix (Yeasen, China).

Thermal cycling was performed on an Applied Biosystems 9700 instrument (Thermo Fisher Scientific, USA), beginning with an initial denaturation at 95 °C for 3 min. This was followed by two-phase amplification: five cycles at 95 °C for 30 s, 45 °C for 30 s, and 72 °C for 30 s, then 20 additional cycles at 95 °C for 30 s, 55 °C for 30 s, and 72 °C for 30 s. A final extension step was conducted at 72 °C for 5 min. Amplification success was verified by 2% (w/v) agarose gel electrophoresis in TBE buffer with ethidium bromide staining, and visualized under UV illumination.

Amplicons were purified using Hieff NGS DNA Selection Beads (Yeasen, China) to remove residual primers and non-specific products. Equimolar pooling of purified PCR products was performed based on DNA concentration measurements, ensuring uniform library representation. Sequencing libraries were constructed by ligating Illumina platform-specific adapters and sample indices, and subsequently sequenced on the MiSeq platform (Illumina, San Diego, CA, USA) according to the manufacturer's standard protocol.

**Text S3. LC–MS/MS-Based Untargeted Metabolomic Profiling**

**HPLC conditions**

Untargeted metabolomic profiling was performed using a liquid chromatography–mass spectrometry (LC–MS) platform operated under automated acquisition sequencing. Chromatographic separation was achieved on a Waters ACQUITY UPLC HSS T3 column (1.8 μm, 2.1 mm × 100 mm) maintained at 40°C. The mobile phase consisted of water with 0.1% formic acid (solvent A) and acetonitrile with 0.1% formic acid (solvent B), delivered at a constant flow rate of 0.40 mL/min. A 4 μL aliquot of each sample was injected into the system.

Gradient elution was applied as follows: the initial condition (95% A, 5% B) was maintained briefly, followed by a linear shift to 35% A and 65% B over 5 min. This was further adjusted to 1% A and 99% B within 1 min and held for 1.5 min. The system was then returned to the starting composition within 0.1 min and equilibrated for an additional 2.4 min.

**MS conditions**

Mass spectrometric detection was carried out in both positive and negative ionization modes using the information-dependent acquisition (IDA) strategy on a Sciex TripleTOF system, controlled by Analyst TF 1.7.1 software (Sciex, Concord, ON, Canada). Ion source settings included: gas 1 (GAS1) at 50 psi, gas 2 (GAS2) at 60 psi, curtain gas (CUR) at 35 psi, and a source temperature of 550 °C. Declustering potential (DP) was set to +80 V for positive mode and –80 V for negative mode. Ion spray voltages (ISVF) were configured at +5500 V and –4500 V for the respective modes.

For time-of-flight (TOF) MS scans, the acquisition parameters were: mass range of 50–1250 Da, accumulation time of 200 ms, and dynamic background subtraction enabled. Product ion scans employed the following settings: mass range of 50–1250 Da, accumulation time of 40 ms, collision energy (CE) set at ±30 V, and collision energy spread of 15 V. Additional MS/MS parameters included: unit resolution, charge state limited to +1, a minimum intensity threshold of 100 counts per second (cps), exclusion of isotopes within 4 Da, a mass tolerance of 50 mDa, and a maximum of 12 candidate ions monitored per cycle.

**Text S4. Growth dynamics of *Microcystis aeruginosa* PCC 7806 under different temperature recovery regimes**

The optical density (OD_720_) of *M. aeruginosa* PCC 7806 cultures exhibited distinct temporal patterns across the three temperature treatments (Figure S1). In G1, OD_720_ increased gradually after day 5, reaching approximately 0.12 by day 20. In G2 , a delayed but more rapid growth phase was observed after day 10, resulting in the highest final OD_720_ (~0.24). In contrast, G3 showed a slower recovery, with OD_720_ reaching only ~0.11 by day 20. These results indicate that temperature recovery strategies influenced *Microcystis* growth kinetics, with gradual warming promoting the most robust biomass accumulation.

**2.** **Supplementary Figures**


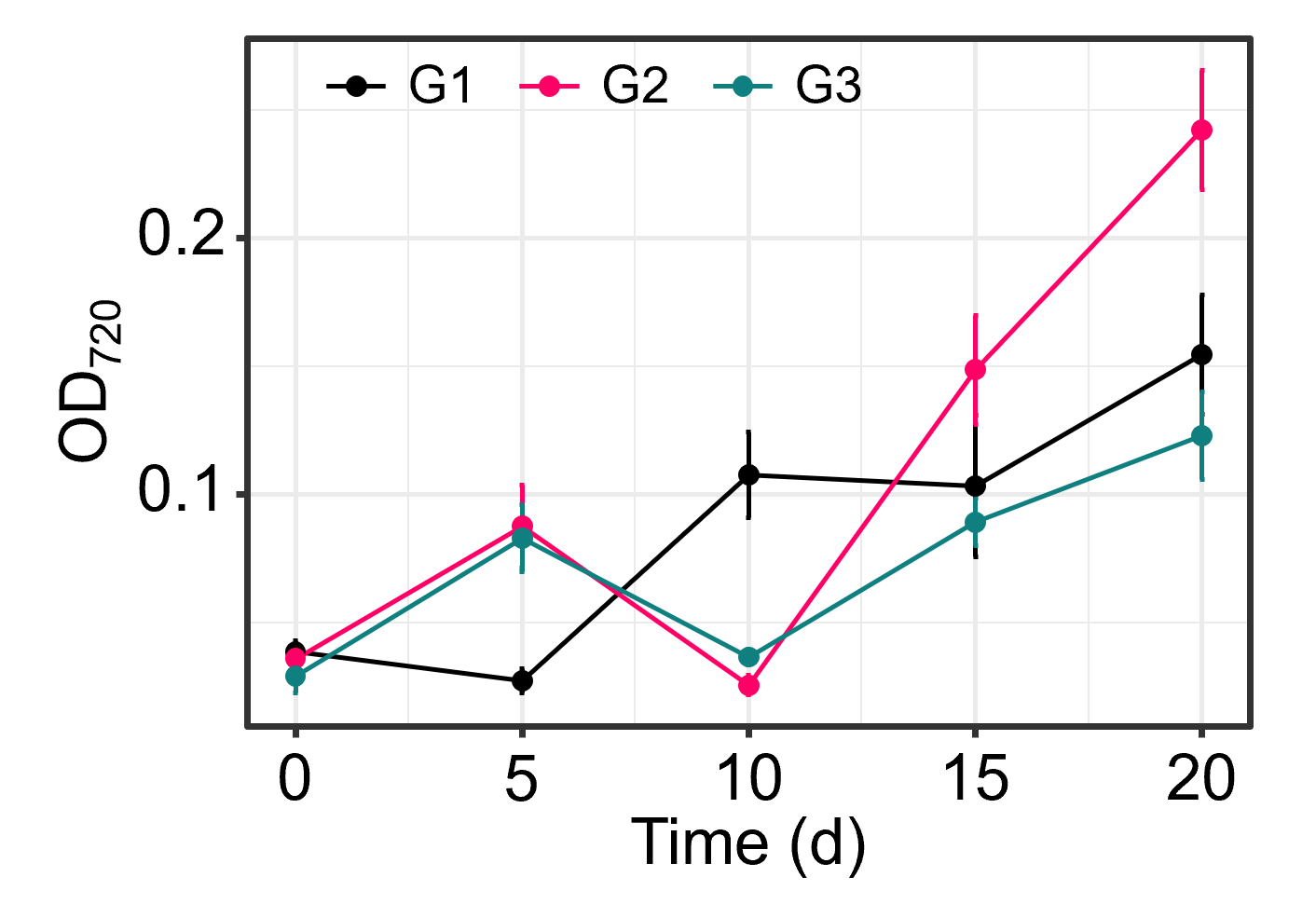


**Figure S1. Growth dynamics of *Microcystis aeruginosa* PCC 7806 under different temperature recovery treatments as indicated by OD_720_ measurements.**


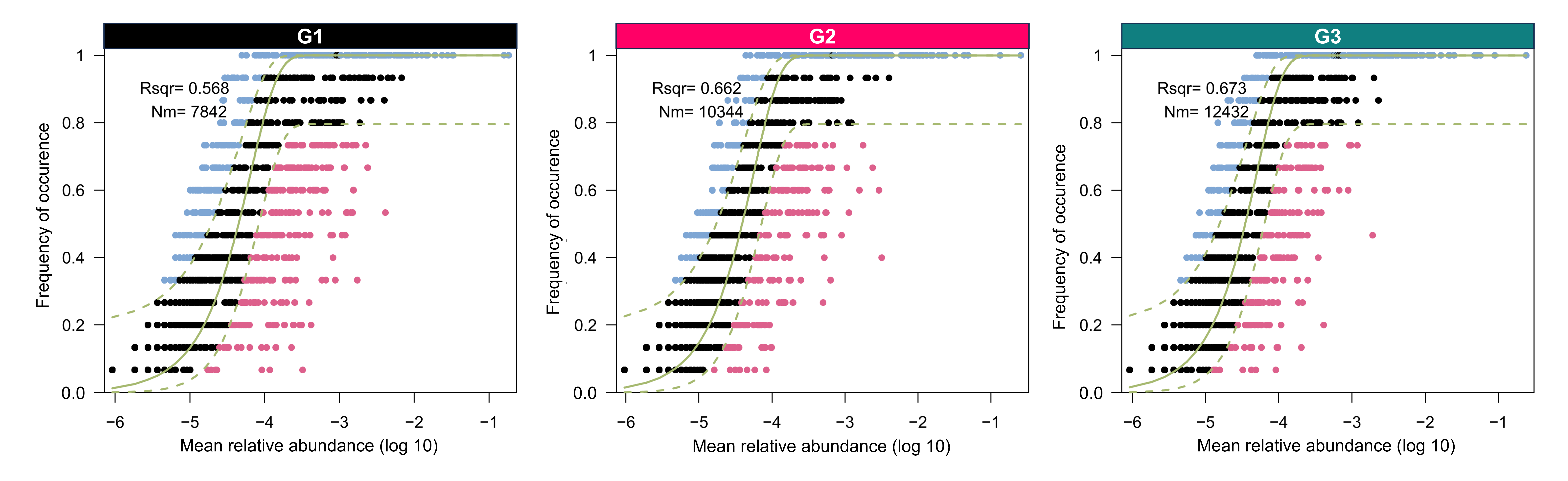


**Figure S2. Neutral community model fitting of bacterial taxa under different treatments.** The coefficient of determination (Rsqr) and total number of OTUs (Nm) are provided for each treatment. Points represent observed taxa; the solid green line indicates the best-fit neutral model prediction, with 95% confidence intervals shown as dashed lines.


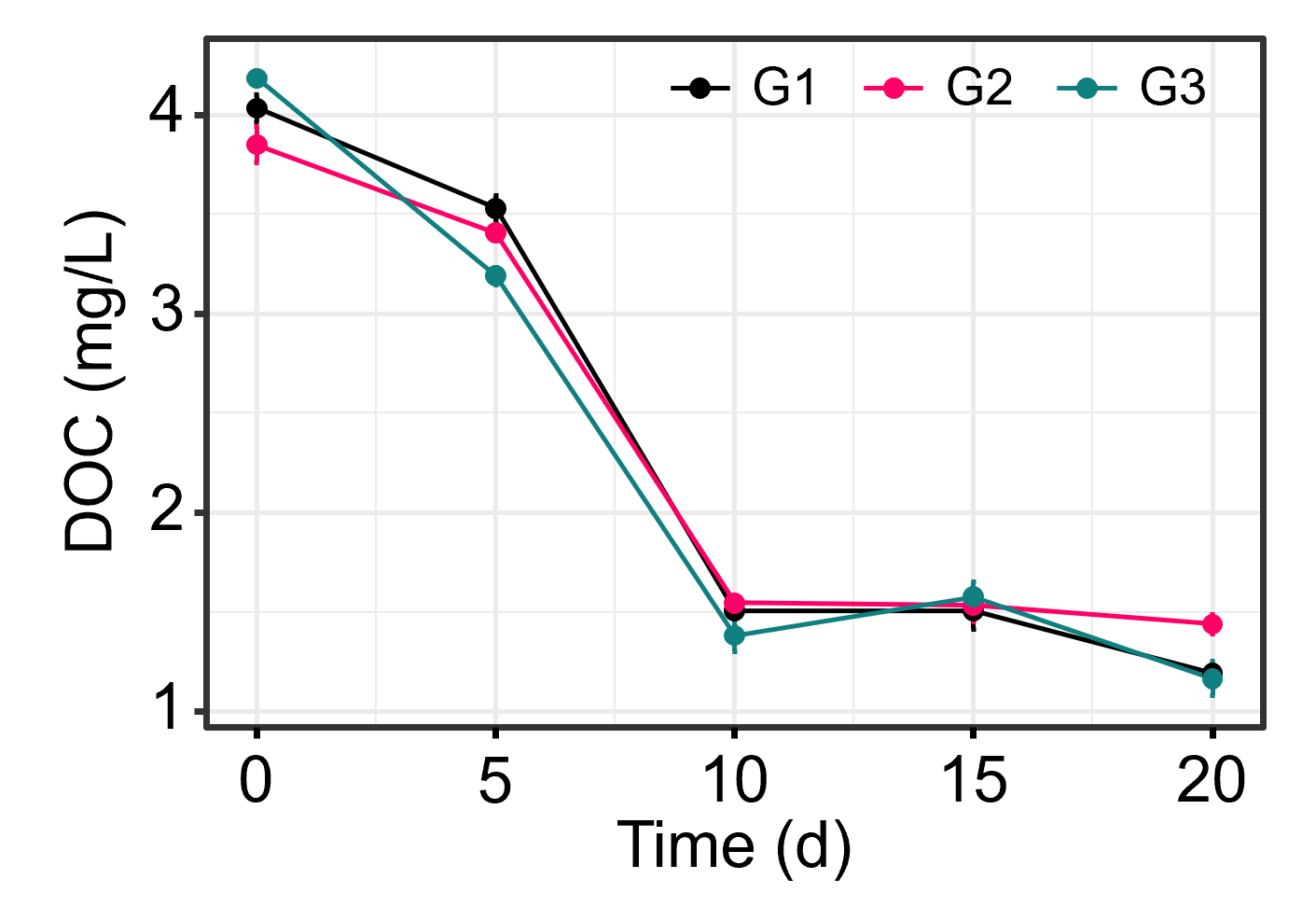


**Figure S3. Temporal variation of DOC concentrations under different temperature recovery treatments.**


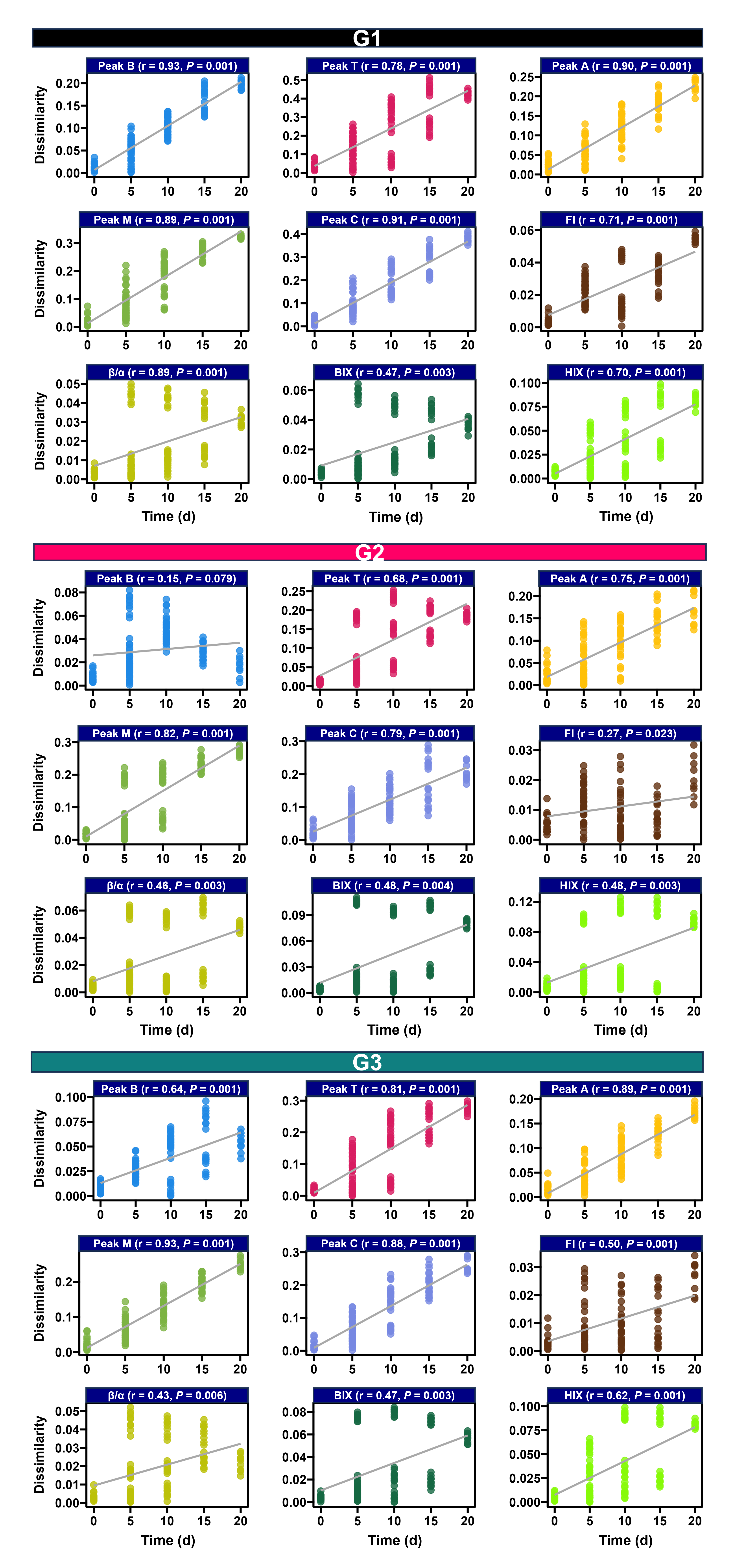


**Figure S4. Temporal patterns of DOM compositional dissimilarity based on fluorescence components and spectral indices under different treatments.** Each plot displays the linear correlation between dissimilarity and incubation time, with corresponding Pearson correlation coefficients (r) and significance levels (*P*).


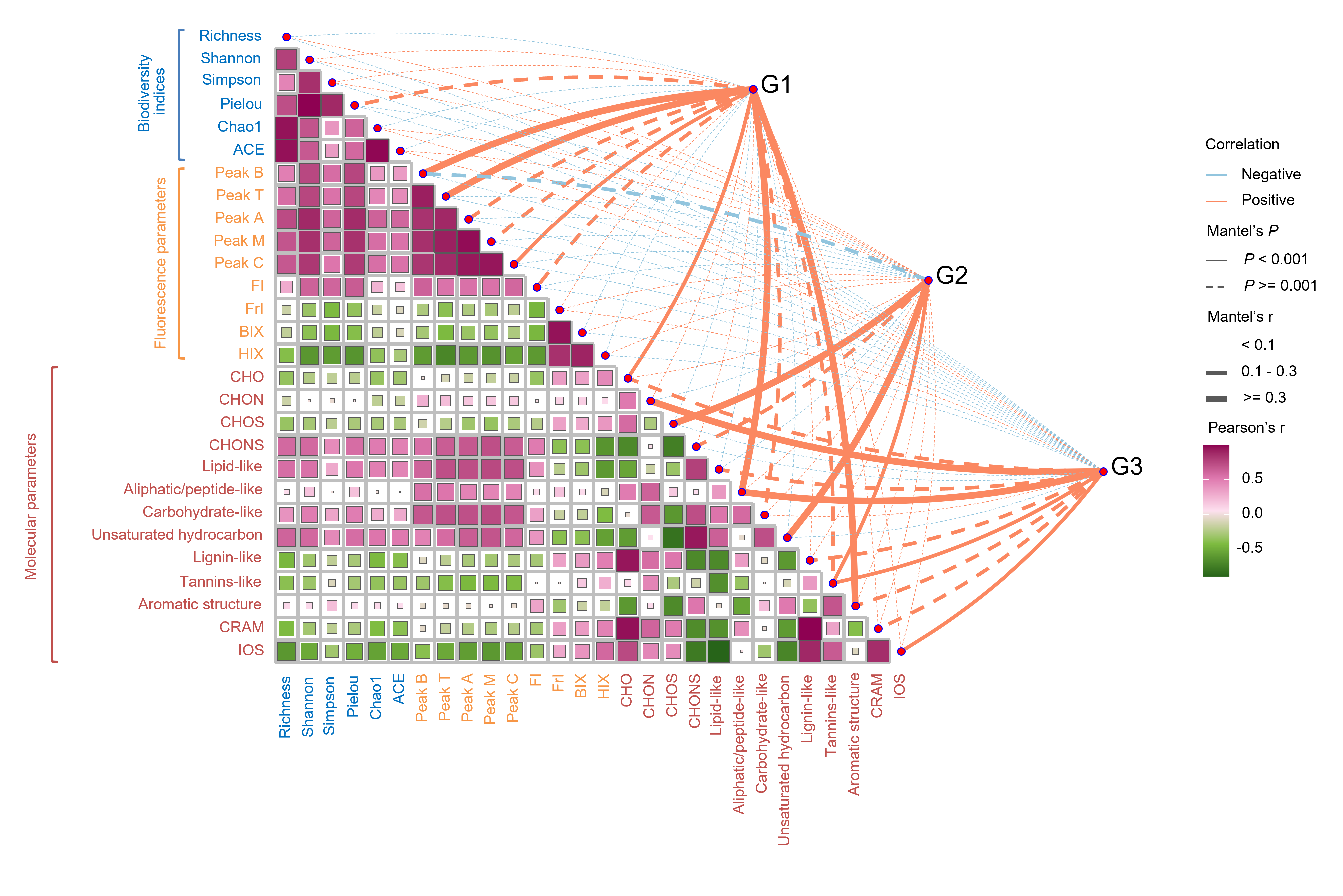


**Figure S5. Integrated correlation network linking bacterial diversity, DOM fluorescence characteristics, and molecular composition across different temperature recovery regimes (G1, G2, G3).Table S1 Water quality parameters of the Fen River in experiment.**

| Water quality parameters | Value (mean ± standard deviation) |
| --- | --- |
| Chemical oxygen demand (COD) | 7.67 ± 0.58 |
| Total phosphorus (TP) | 0.02 ± 0.01 |
| Ammonium-nitrogen (NH_4_–N) | 0.56 ± 0.04 |
| Nitrate-nitrogen (NO_3_–N) | 0.12 ± 0.03 |
| Dissolved organic carbon (DOC) | 2.02 ± 0.06 |

**Table S2 Identification and literature comparison of spectral properties for five fluorescent peaks.**

| Fluorescent peak | Ex/Em | Description | Probable source | Reference |
| --- | --- | --- | --- | --- |
| Peak B | 250 (275)/305 nm | Tyrosine-like | Autochthonous | [3] |
| Peak T | 250 (275)/330-340 nm | Tryptophan-like | Autochthonous | [3, 4] |
| Peak A | 250/370-420 nm | Humic-like | Terrestrial | [5] |
| Peak C | 340/400 nm  360/460 nm | Humic-like | Terrestrial/Autochthonous | [6] |
| Peak M | 290/380 nm | Humic-like | Microbial processing of organic matter | [7] |

**Table S3 Formula for calculating DOM molecules.**

| DOM molecular | Equation | Reference |
| --- | --- | --- |
| Nominal oxidation state of carbon (NOSC) | $NOSC=4-\frac{4C+H-3N-2O-2S}{C}$ | [8] |
| Modified aromaticity index (AI_mod_) | $AI_{\text{mod}}=\frac{1+2C+0.5O-S-0.5(N-P-H)}{C-0.5O-S-N-P}$ | [9, 10] |
| Double bond equivalents (DBE) | $DBE=\frac{2C-H+N+P+2}{2}$ | [9] |
| Gibbs free energy (△*G*^◦^_Cox_) | $\text{△}G^{\text{o}}_{\text{Cox}}=-28.5\times NOSC+60.3$ | [8] |
| Carboxylic-rich alicyclic compounds (CRAM) | Formulas (DBE/C = 0.3–0.68, DBE/H = 0.2–0.95, and DBE/O = 0.77–1.75). | [11] |
| Island of stability (IOS) | Formulas (H/C = 1.17 ± 0.13, O/C = 0.52 ± 0.10) and molecular mass (360 ± 28 and 497 ± 51 Da). | [12] |

**Table S4. Gene abbreviations and annotations.**

| Gene | Description |
| --- | --- |
| *fabG* | 3-oxoacyl-[acyl-carrier protein] reductase [EC:1.1.1.100] |
| *gst* | glutathione S-transferase [EC:2.5.1.18] |
| *ABC.PE.S* | peptide/nickel transport system substrate-binding protein |
| *livK* | branched-chain amino acid transport system substrate-binding protein |
| *ABC.PE.A1* | peptide/nickel transport system ATP-binding protein |
| *livF* | branched-chain amino acid transport system ATP-binding protein |
| *livH* | branched-chain amino acid transport system permease protein |
| *livM* | branched-chain amino acid transport system permease protein |
| *livG* | branched-chain amino acid transport system ATP-binding protein |
| *pstS* | phosphate transport system substrate-binding protein |
| *atoB* | acetyl-CoA C-acetyltransferase [EC:2.3.1.9] |
| *dcm* | DNA (cytosine-5)-methyltransferase 1 [EC:2.1.1.37] |
| *clpP* | ATP-dependent Clp protease, protease subunit [EC:3.4.21.92] |
| *purL* | phosphoribosylformylglycinamidine synthase [EC:6.3.5.3] |
| *ilvB* | acetolactate synthase I/II/III large subunit [EC:2.2.1.6] |
| *glnA* | glutamine synthetase [EC:6.3.1.2] |
| *mcp* | methyl-accepting chemotaxis protein |
| *gatA* | aspartyl-tRNA(Asn)/glutamyl-tRNA(Gln) amidotransferase subunit A [EC:6.3.5.6 6.3.5.7] |
| *pyrC* | dihydroorotase [EC:3.5.2.3] |
| *amiABC* | N-acetylmuramoyl-L-alanine amidase [EC:3.5.1.28] |
| *dnaK* | molecular chaperone DnaK |
| *trxA* | thioredoxin 1 |
| *ggt* | gamma-glutamyltranspeptidase / glutathione hydrolase [EC:2.3.2.2 3.4.19.13] |
| *dapA* | 4-hydroxy-tetrahydrodipicolinate synthase [EC:4.3.3.7] |
| *galE* | UDP-glucose 4-epimerase [EC:5.1.3.2] |
| *suhB* | myo-inositol-1(or 4)-monophosphatase [EC:3.1.3.25] |
| *iscS* | cysteine desulfurase [EC:2.8.1.7] |
| *cysK* | cysteine synthase A [EC:2.5.1.47] |
| *serA* | D-3-phosphoglycerate dehydrogenase / 2-oxoglutarate reductase [EC:1.1.1.95 1.1.1.399] |

**Figure S5. Topological properties of bacterial co-occurrence networks under different treatments.**

|  | Average clustering coefficient | Average degree | Network diameter | Modularity | Node | Edge | Density | Positive connections | Negative connections |
| --- | --- | --- | --- | --- | --- | --- | --- | --- | --- |
| G1 | 0.43 | 11.94 | 14 | 0.42 | 368 | 2197 | 0.03 | 87.80% | 12.20% |
| G2 | 0.50 | 17.52 | 10 | 0.25 | 281 | 2462 | 0.06 | 74.05% | 25.95% |
| G3 | 0.55 | 23.74 | 10 | 0.28 | 278 | 3300 | 0.09 | 68.64% | 31.36% |

**Figure S6. Relative contributions of network modules in bacterial co-occurrence networks under different treatments.**

| Moudle | M1 | M2 | M3 | M4 | M5 | M6 | M7 | M8 | M9 | M10 |
| --- | --- | --- | --- | --- | --- | --- | --- | --- | --- | --- |
| G1 | 36.41% | 28.26% | 18.21% | 14.95% | 0.54% | 0.54% | 0.54% | 0.54% | / | / |
| G2 | 56.94% | 23.49% | 14.59% | 1.07% | 1.07% | 0.71% | 0.71% | 0.71% | 0.71% | / |
| G3 | 28.06% | 26.62% | 21.94% | 18.71% | 1.08% | 0.72% | 0.72% | 0.72% | 0.72% | 0.72% |

**Table S7. Proportional distribution of DOM molecular formulas in thermodynamic space defined by NOSC and (DBE–O)/C under different treatments.**

|  |  | I (unsaturated and reduced compounds) (%) | II (unsaturated and oxidized compounds) (%) | III (saturated and reduced compounds) (%) | IV (saturated and oxidized compounds) (%) |
| --- | --- | --- | --- | --- | --- |
| G1 | Day 0 | 25.75 | 6.84 | 50.64 | 7.17 |
|  | Day 20 | 24.96 | 7.08 | 50.79 | 7.31 |
| G2 | Day 0 | 25.00 | 7.76 | 49.92 | 7.96 |
|  | Day 20 | 25.76 | 8.26 | 48.15 | 8.62 |
| G3 | Day 0 | 25.13 | 8.96 | 46.99 | 9.23 |
|  | Day 20 | 26.40 | 7.60 | 48.74 | 7.43 |

**Table S8 The results of differential metabolites in the G2 vs G1 group.**

| Index | Compounds | Formula | VIP | *P* value | Log_2_FC | Type |
| --- | --- | --- | --- | --- | --- | --- |
| MW0054292 | Lauroyl diethanolamide | C_16_H_33_NO_3_ | 1.37 | 0.40 | 6.53 | up |
| MW0106770 | gamma-Glutamylglutamate | C_10_H_16_N_2_O_7_ | 1.75 | 0.11 | 6.80 | up |
| MW0055375 | Nostoxanthin | C_40_H_56_O_4_ | 1.75 | 0.05 | 6.11 | up |
| MW0053496 | Geranylgeraniol | C_20_H_34_O | 1.36 | 0.38 | 6.92 | up |
| MW0148473 | Digitalin | C_36_H_56_O_14_ | 1.75 | 0.05 | 6.05 | up |
| MW0153001 | Lys-Ile-Arg-Asp | C_22_H_42_N_8_O_7_ | 1.36 | 0.32 | 6.69 | up |
| MW0156981 | Ser-Val-Lys-Arg | C_20_H_40_N_8_O_6_ | 1.58 | 0.26 | 6.38 | up |
| MW0011866 | 1,2-Docosahexanoyl-sn-glycero-3-phosphocholine | C_52_H_80_NO_8_P | 1.66 | 0.16 | 5.75 | up |
| MW0156666 | Ser-Asn-Ser | C_10_H_18_N_4_O_7_ | 1.51 | 0.17 | 6.07 | up |
| MW0006648 | Convolamine | C_17_H_23_NO_4_ | 1.71 | 0.21 | 7.24 | up |

**Table S9 Results of differential metabolites in the G3 vs G1 group.**

| Index | Compounds | Formula | VIP | *P* value | Log_2_FC | Type |
| --- | --- | --- | --- | --- | --- | --- |
| MW0107956 | L-Naspa | C_19_H_38_NO_7_P | 1.29 | 0.37 | 5.50 | up |
| MW0106770 | gamma-Glutamylglutamate | C_10_H_16_N_2_O_7_ | 1.72 | 0.33 | 8.26 | up |
| MW0055375 | Nostoxanthin | C_40_H_56_O_4_ | 1.57 | 0.23 | 6.25 | up |
| MW0145335 | Arg-Gln-Tyr-Lys | C_26_H_43_N_9_O_7_ | 1.11 | 0.37 | 5.72 | up |
| MW0145433 | Arg-Lys-Asp-Lys-Glu | C_27_H_50_N_10_O_10_ | 1.40 | 0.38 | 5.50 | up |
| MW0157706 | Thr-Leu-Lys-Lys | C_22_H_44_N_6_O_6_ | 1.59 | 0.18 | 5.72 | up |
| MW0011866 | 1,2-Docosahexanoyl-sn-glycero-3-phosphocholine | C_52_H_80_NO_8_P | 1.81 | 0.05 | 6.24 | up |
| MW0156666 | Ser-Asn-Ser | C_10_H_18_N_4_O_7_ | 1.39 | 0.21 | 6.82 | up |
| MW0104538 | 2-amino-4-({1-[(carboxymethyl)-C-hydroxycarbonimidoyl]-2-[(1-hydroxy-5-oxo-1,7-diphenylheptan-2-yl)sulfanyl]ethyl}-C-hydroxycarbonimidoyl)butanoic acid | C_29_H_37_N_3_O_8_S | 1.66 | 0.24 | 5.65 | up |
| MW0006648 | Convolamine | C_17_H_23_NO_4_ | 1.52 | 0.40 | 8.39 | up |

**References**

1. Crouch SR, Malmstadt HV. Mechanistic investigation of molybdenum blue method for determination of phosphate. Anal Chem 1967;39:1084-9.
2. Wood ED, Armstrong FAJ, Richards FA. Determination of nitrate in sea water by cadmium-copper reduction to nitrite. J Mar Biol Assoc UK 1967;**47**:23-31.
3. Coble PG, Del Castillo CE, Avril B. Distribution and optical properties of CDOM in the Arabian Sea during the 1995 Southwest Monsoon. Deep Sea Res Part II 1998;**45**:2195-223. <https://doi.org/>10.1016/S0967-0645(98)00068-X
4. Coble PG. (1996). Characterization of marine and terrestrial DOM in seawater using excitation-emission matrix spectroscopy. Mar Chem 1996;**51**:325-46. https://doi.org/10.1016/0304-4203(95)00062-3
5. Stedmon CA, Markager S, Bro R. Tracing dissolved organic matter in aquatic environments using a new approach to fluorescence spectroscopy. Mar Chem 2003;**82**:239-54. <https://doi.org/>10.1016/s0304-4203(03)00072-0
6. Williams CJ, Yamashita Y, Wilson HF et al. Unraveling the role of land use and microbial activity in shaping dissolved organic matter characteristics in stream ecosystems. Limnol Oceanogr 2010;**55**:1159-71. <https://doi.org/>10.4319/lo.2010.55.3.1159
7. Murphy KR, Stedmon CA, Waite TD et al. Distinguishing between terrestrial and autochthonous organic matter sources in marine environments using fluorescence spectroscopy. Mar Chem 2008;**108**:40-58. https://doi.org/10.1016/j.marchem.2007.10.003
8. LaRowe DE, Van Cappellen P. Degradation of natural organic matter: a thermodynamic analysis. Geochim Cosmochim Acta 2011;**75**:2030-42. <https://doi.org/10.1016/j.gca.2011.01.020>
9. Koch BP, Dittmar T. From mass to structure: an aromaticity index for highresolution mass data of natural organic matter. Rapid Commun Mass Spectrom 2006;**20**:926-32. <https://doi.org/10.1002/rcm.2386>
10. Koch, BP, Dittmar T. From mass to structure: an aromaticity index for highresolution mass data of natural organic matter (vol 20, pg 926, 2006). Rapid Commun Mass Spectrom 2016;**30**:250. <https://doi.org/10.1002/rcm.7433>
11. Lechtenfeld OJ, Hertkorn N, Shen Y, et al. Marine sequestration of carbon in bacterial metabolites. Nat Commun 2015;**6**:1-8. <https://doi.org/10.1038/ncomms7711>
12. Lechtenfeld OJ, Kattner G, Flerus R et al. Molecular transformation and degradation of refractory dissolved organic matter in the Atlantic and Southern Ocean. Geochim Cosmochim Acta 2014;**126**:321-337. <https://doi.org/10.1016/j.gca.2013.11.009>
